# Cellular multipoint adaptive technology for two-photon mesoscope

**DOI:** 10.1117/1.NPh.13.1.015004

**Published:** 2026-01-20

**Authors:** Shuai Chen, Mengke Yang, Jing Lyu, Yu Huang, Hong Ye, Qian Gao, Tangmin Chen, Haiyang Chen, Huanhuan Zeng, Min Li, Yunyun Han, Xiaowei Chen, Zsuzsanna Varga, Arthur Konnerth, Zhenqiao Zhou, Yuguo Tang, Hongbo Jia

**Affiliations:** a Guangxi University, Advanced Institute for Brain and Intelligence and School of Physical Science and Technology, Nanning, China; b Chinese Academy of Sciences, Suzhou Institute of Biomedical Engineering and Technology, Jiangsu Key Laboratory for Advanced Theranostics and Medical Instrumentation, Suzhou, China; c Leibniz Institute for Neurobiology (LIN), Magdeburg, Germany; d University of Oxford, Department of Pharmacology, Oxford, United Kingdom; e Huazhong University of Science and Technology, Tongji Medical College, School of Basic Medicine, Department of Neurobiology, Wuhan, China; f Chinese Academy of Sciences, Institute of Genetics and Developmental Biology, State Key Laboratory of Molecular Developmental Biology, Beijing, China; g Lingang Laboratory, Shanghai, China; h Chongqing Institute for Brain and Intelligence, Guangyang Bay Laboratory, Chongqing, China; i Technical University of Munich, Institute of Neuroscience and the SyNergy Cluster, Munich, Germany

**Keywords:** two-photon mesoscope, adaptive optics, real-time wavefront correction

## Abstract

**Significance:**

In mesoscopic imaging research in neuroscience, achieving high spatial resolution optical imaging across the entire field of view (FOV) remains critical. This directly determines whether researchers can precisely analyze the large-scale dynamic activities of neural circuits at the single-cell or even subcellular level. Consistent optical quality throughout the entire imaging FOV is essential to accurately capture the spatiotemporal patterns of neural activity across brain regions, thereby providing a powerful tool for understanding the circuit mechanisms underlying cognition, behavior, and disease at cellular and subcellular resolution *in vivo*.

**Aim:**

This study aims to develop a technology that extends the imaging FOV in a two-photon mesoscope while enhancing the optical quality across the entire FOV *in vivo*. The key point is to establish a robust method that can significantly extend the FOV beyond what the micro/mesoscope objective had been originally designed for, yet maintain the original resolution specifications. As such, the value of the method also extends beyond improving just one mesoscope, which we use as a demo in this study.

**Approach:**

This study introduces an innovative approach that combines block scanning with adaptive optical (AO) correction through a bioinspired honeycomb-based cellular multipoint adaptive technology (CMAT) to achieve mesoscopic two-photon imaging. This system enables unprecedented large-FOV, high-resolution imaging by dividing an $8\times 8\;{\mathrm{mm}}^{2}$ imaging area into subregions, each pre-optimized with deformable mirror (DM) compensation while applying real-time dynamic wavefront correction during scanning. Furthermore, we have designed multiple user-defined sub-region scanning functions. Each sub-region automatically loads the aberration correction compensation values from the nearest reference point relative to its center, thereby ensuring optimal optical performance for every individual sub-region. The robustness of this technology has been systematically verified across multiple neural circuit observation scenarios using transgenic mouse models, demonstrating its capability for reliable single-cell resolution imaging across extensive brain regions.

**Results:**

Comprehensive evaluation using standard samples and transgenic mouse models demonstrated that the CMAT significantly enhances the imaging performance of the two-photon mesoscope. This technique extends the effective two-photon imaging FOV from $6\times 6\;mm^{2}$ to $8\times 8\;mm^{2}$ while markedly improving the optical quality in the peripheral regions. High resolution was maintained at ∼1  μm (lateral) and ∼10  μm (axial) in the central area, with edge regions achieving improved resolutions of ∼1.3  μm (lateral) and ∼14  μm (axial). Quantitative analysis confirmed that multipoint AO not only enhances image contrast and optical resolution but also substantially increases the signal-to-noise ratio (SNR) in ${\mathrm{Ca}}^{2+}$ imaging. This work delivers a pivotal technical advance for large-scale functional imaging of neural circuits.

**Conclusion:**

CMAT significantly extends the effective FOV and enhances the optical quality of the two-photon mesoscope system.

## 1 Introduction

Two-photon fluorescence microscopy has played an important role in neuroscience research because its inception, owing to its unique optical properties.^1^^–^^3^ By utilizing near-infrared femtosecond laser-based two-photon excitation, this technology achieves unparalleled tissue penetration depths of 500 to 1000  μm, enabling noninvasive access to subcortical layers while maintaining exceptional optical sectioning capability. Compared with conventional widefield or confocal microscopy, two-photon imaging offers three major advantages for *in vivo* studies: (1) reduced scattering of near-infrared excitation light enables deep-tissue imaging; (2) nonlinear excitation confines fluorescence generation to the focal volume, significantly enhancing three-dimensional (3D) resolution; and (3) longer-wavelength excitation causes substantially less phototoxicity, making it ideal for long-term *in vivo* observations.^4^^–^^12^

However, existing two-photon microscopy systems still face critical challenges in achieving large-scale and high-resolution imaging simultaneously. Traditional systems typically offer a limited field of view (FOV) below $1\;mm^{2}$, which falls short of the requirements for whole-cortex-scale neural circuit studies and hinders comprehensive investigations of distributed neural network activity.^13^^–^^16^

According to the optical invariant theorem, microscope objectives present a trade-off between the FOV and numerical aperture (NA).^17^^–^^19^ At the FOV edge, off-axis aberrations, e.g., astigmatism and coma, become significantly pronounced. These aberrations degrade the point spread function and reduce the fluorescence excitation efficiency.^20^^,^^21^ Such a degradation is even more severe in two-photon imaging because of the nonlinear excitation relationship. Currently, commercial two-photon mesoscopes typically offer FOV between 3×3 and $5\times 5\;mm^{2}$, i.e., 9 to $25\;mm^{2}$. The expansion of FOV is fundamentally limited by the objective optics and its aberration correction capability, leading to a drastic decline in imaging quality at the edge of FOV, and thus raising a critical obstacle on the path toward precise whole-cortex neuronal activity mapping.^22^^–^^26^

Although adaptive optics (AO) has demonstrated excellent aberration correction ability in biomedical imaging, its application at mesoscopic scales poses unique challenges. Conventional AO methods rely on a single wavefront compensation and are difficult to correct for the entire FOV effectively; local correction leaves residual aberrations in other regions, whereas global averaging fails to achieve optimal correction.^27^^–^^29^ Recent studies suggest that partition-based correction strategies could address this issue.^30^^,^^31^ For instance, dividing a $450\times 450\;\mu m^{2}$ FOV into nine subregions of $150\times 150\;\mu m^{2}$ for parallel compensation can remarkably improve the image quality.^32^ However, this multiregion AO approach has not yet been applied to the mesoscopic imaging with an FOV larger than $5\times 5\;mm^{2}$. The significant increase in FOV leads to pronounced spatial heterogeneity in the types and magnitudes of aberrations between the peripheral and central regions of the FOV. Such irregular distribution makes the development of multiregion adaptive optics technology for millimeter-scale FOV systems a key challenge in improving mesoscopic imaging performance.

To correct aberrations at the edge of the FOV in the two-photon mesoscope, this work introduces an innovative method: cellular multipoint adaptive technology (CMAT), based on the Ultra-wide-field, deep, adaptive two-photon microscopy (ULTRA) mesoscope system.^33^ By constructing multiple hexagonally tiled correction zones across the entire FOV and deploying equidistant compensation points at each vertex and geometric center, we have achieved comprehensive full-FOV aberration correction. Experimental results show that CMAT not only extends the effective imaging FOV from Ø6 to Ø8 mm in diameter, i.e., twice the imaging area but also notably improves the imaging performance at the edge of the FOV. This multipoint correction strategy provides a novel approach to overcome aberration limitations in mesoscopic imaging, advancing the full-FOV optical performance closer to the diffraction limit.

## 2 Method

### 2.1 Two-photon Mesoscope with CMAT

The experimental setup employs a femtosecond laser system (Spark Laser, ALCOR 920-4-XSight, Martillac, 33650, France) operating at a center wavelength of 920 nm, with a repetition rate of 80 MHz, output power of ∼3.4  W, and pulse width of ∼130  fs. The scanning module consists of an 8 kHz resonant mirror (CRS8k, Cambridge Technology, Massachusetts, United States) for fast-axis scanning, and a pair of galvanometers (Galvos, Newton, New Jersey, United States) (QS20XY-AG, Thorlabs, Newton, New Jersey, United States) for slow-axis positioning. Wavefront correction was achieved using a 97-actuator deformable mirror (DM97-15, ALPAO, Montbonnot Saint-Martin, France) with a 0.8 ms response time and 13.5 mm input pupil diameter. A custom-designed water-immersion objective (NA = 0.5) was used to have a FOV of Ø6 mm with diffraction-limited resolution at a working distance of 2 mm, paired with custom-built large-aperture 4f relay optics. The central control system is built on a PXI platform (PXIe‐1082, National Instruments,Texas, United States) utilizing an FPGA multifunction card (PXI‐7851R, National Instruments, Texas, United States), which provides both the Galvos scanning waveforms and the reference trigger for data acquisition. Fluorescence signals were detected by a GaAsP PMT (H15460-40, Hamamatsu, Hamamatsu City, Japan) with a detection area of 14  mm×14  mm and were acquired using a high-resolution PXI oscilloscope (PXIe‐5122, National Instruments; 100 MHz sampling rate, Texas, United States).

**Fig. 1:**
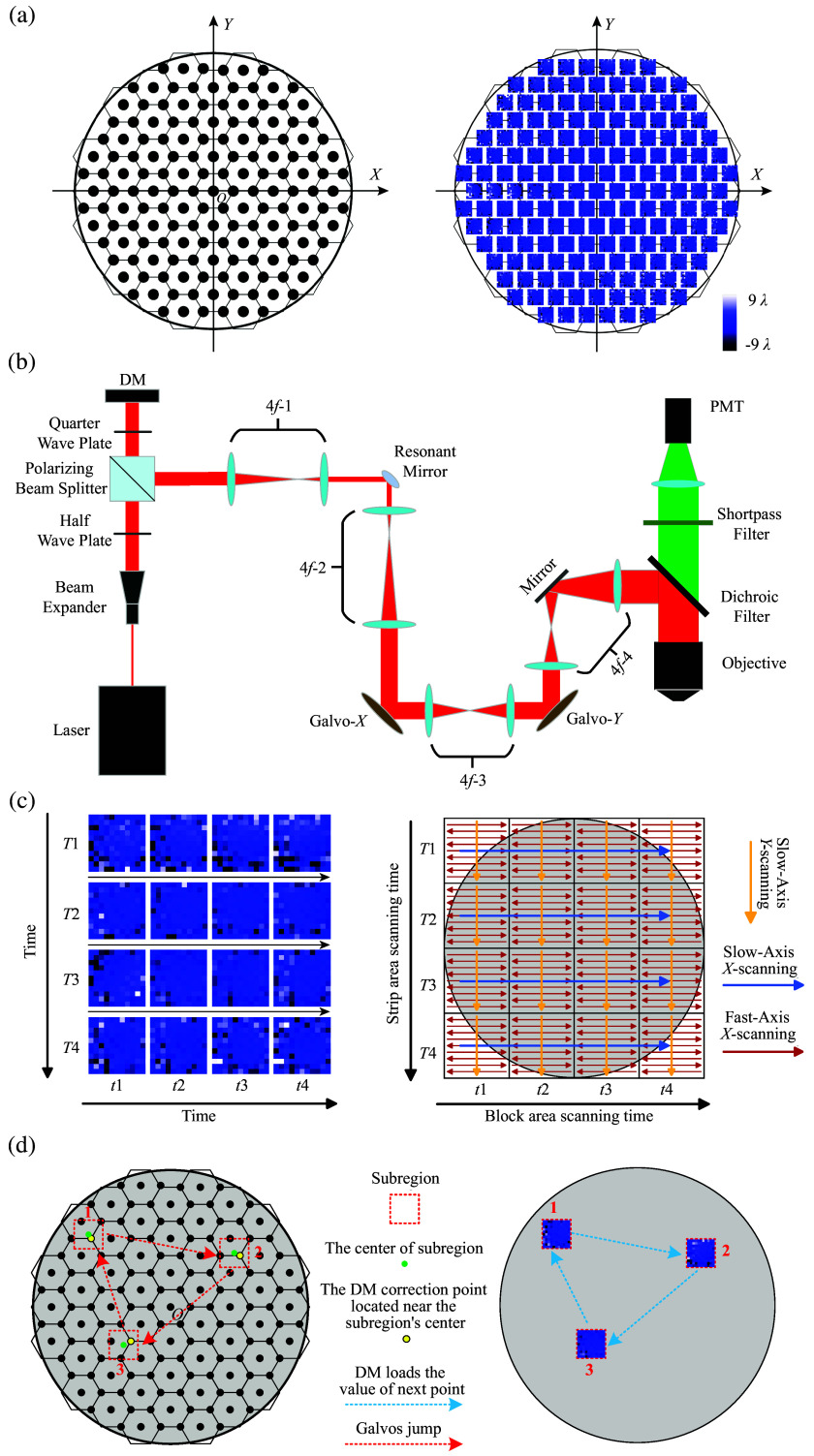
Principles of CMAT. (a) Measurement points within the full FOV and corresponding phase maps for all measurement points. (b) Schematic diagram of the optical setup. (c) Timing diagram of object-plane scanning positions and DM phase modulation: yellow indicates the slow-axis Galvo-Y scanning direction, blue represents the slow-axis Galvo-X scanning direction, and red shows the fast-axis resonant mirror scanning direction. The object plane is divided into 4×4 scanning regions. For each scanned sub-region, the DM loads a corresponding phase map, totaling 16 phase modulations per full-FOV scan. The scanning sequence proceeds left-to-right: the resonant mirror and Galvo-Y first complete a sub-region scan, then Galvo-X shifts rightward until four sub-regions are scanned to form a stripe. Subsequently, Galvo-X and Galvo-Y reposition to initiate the next stripe scan. This process repeats four times to accomplish a complete full-FOV scan. (d) Schematic of the ROI function. Red frames delineate the scanning subregions. Green points mark the centroids of these subregions, whereas yellow points indicate the corresponding DM correction points. Red arrow-headed lines represent the Galvos jump trajectories, and blue arrow-headed lines signify the loading of optical correction values for the subsequent target points. The Galvos jump and the loading of optical correction values are executed concurrently.

The purpose of CMAT is to correct optical aberrations induced by the microscope system by applying pixel-based wavefront correction throughout the entire mesoscopic FOV. Hexagonal tiling was used to optimize the division of the full FOV. As shown in Fig. 1(a), the tiling pattern originates from point O (the FOV center), which coincides with the center of the first hexagon, extending outward to fill the FOV with a total of 55 hexagons. Using both the vertices and centers of these hexagons, we established a uniform grid of 163 correction/compensation points. The corresponding control voltages for the Galvo system were then calculated based on the coordinates of these points. As shown in Fig. S1(a) in the Supplementary Material, measurements were taken at multiple positions (1, 2, 3, and 3.6 mm from the center) across the full FOV. It was observed that the optical resolution in peripheral regions declined with increasing distance from the compensation point, as evaluated by the Mann–Whitney test [see Figs. S1(b) and S1(e) in the Supplementary Material]. This degradation became increasingly apparent beyond 2 mm from the center. Near the edge of the FOV (3.6 mm), there was a sharp drop in optical resolution as the distance from the compensation point increased [see Fig. S1(e) in the Supplementary Material]. Nevertheless, based on experimental results and practical considerations, the degradation at these peripheral positions remains below 10% relative to the center, and the image quality still meets basic imaging standards. To balance image quality and time efficiency—by ensuring every point within the FOV is within an effective compensation range (a radius of 330  μm from a compensation point)—a regular hexagon with a side length of 577  μm was chosen as the basic unit. Its center and vertices act as aberration compensation points. This side length was selected because the hexagon’s center and any two adjacent vertices form an equilateral triangle, and the circumcenter of this triangle is roughly 330  μm from each vertex.

The pollen grain sample was employed for the wavefront correction calibration.^34^ Before the calibration, the sample was placed in the center of the FOV. When pollen samples were utilized for system aberration correction, variations in the higher-order Zernike coefficients were observed to influence the fluorescence intensity. Moreover, analysis of the exported coefficients indicated that these higher-order terms varied at each correction point. Consequently, the correction of higher-order aberrations was upheld throughout the procedure [see Fig. S2(a) in the Supplementary Material]. The correction procedure involved sequential optimization of the first 200 Zernike coefficients. According to the specifications of the deformable mirror (DM), the coefficients were multiplied by the corresponding Zernike polynomials for each term to execute the actuator control. Meanwhile, for each Zernike term, the correction was performed over five iterative cycles, with 21 steps per cycle, within the predetermined coefficient range. Throughout this process, the optimization was guided by maximizing the fluorescence intensity from the pollen sample, with the Zernike coefficient corresponding to the maximum intensity at each cycle being identified and recorded. The peak values from each cycle were averaged and used as the final coefficient for the corresponding Zernike term. The same process was repeated sequentially for all 200 Zernike coefficients, constituting one complete correction round. The calibration typically requires 4 to 8 correction rounds to achieve optimal compensation value for each correction point, ensuring precise wavefront correction across the full range of spatial frequencies relevant to the imaging system. The final wavefront map loaded is normalized relative to the DM’s stroke range, which is ±30 λ.

In this work, we introduce a hybrid scanning system that combines a high-speed resonant mirror (fast-axis scanner) with a Galvo mirror pair (slow-axis scanner) to achieve both rapid imaging and precise positioning [Fig. 1(b)]. The slow-axis Galvos provide a 45 deg maximum scanning angle, sufficient to cover an 8 mm FOV, though their line rate is limited to a few hundred Hz. In contrast, the fast-axis resonant mirror operates at 8 kHz, i.e.,16 kHz bidirectional line rate, with a 26 deg scanning angle, covering a 2.5 mm stripe width. A trade-off exists between scanning speed and image quality in our tiled scanning approach for full-FOV imaging [Fig. 1(c)]. This work employs a 4×4 grid (16 subregions) to maximize scanning speed. Although using a finer grid (e.g., 5×5, 6×6, up to N×N) enhances resolution, it consequently leads to a dramatic increase in the total scan duration. The resonant mirror (X-axis) and one Galvo (Y-axis) first scan a $2\times 2\;mm^{2}$ subregion. The second Galvo (X-axis) then steps to three adjacent positions, sequentially scanning additional subregions to form an $8\times 2\;mm^{2}$ stripe. The process repeats while the resonance mirror scans continuously, with the two Galvos repositioning between stripes until 16 subregions of $2\times 2\;mm^{2}$ arranged in a 4×4 matrix collectively cover the entire FOV. During system operation, the DM automatically retrieves and applies calibrated compensation values corresponding to the nearest correction point relative to each subregion center. This localized adaptive optics approach enables 16 independent wavefront corrections per frame, ensuring complete FOV coverage with optimized optical performance. In addition, as illustrated in Fig. 1(d), the system enables region-of-interest (ROI) scanning capability within the full $8\times 8\;mm^{2}$ FOV. By computing Galvo drive voltages based on ROI centroid coordinates and applying the geometrically nearest pre-calibrated compensation values, the system allows targeted high-speed imaging of user-specified areas. During the Galvos jump, the DM simultaneously loads the aberration compensation values for the next subregion. These two procedures are conducted in parallel and are completed simultaneously. The data collected during this interval is subsequently excluded from the image reconstruction process. This scanning approach is predominantly employed in this study because the sequential scanning of multiple small subregions achieves an optimal balance between image quality and acquisition speed, rendering it especially advantageous for brain function research.

### 2.2 System Resolution

**Fig. 2:**
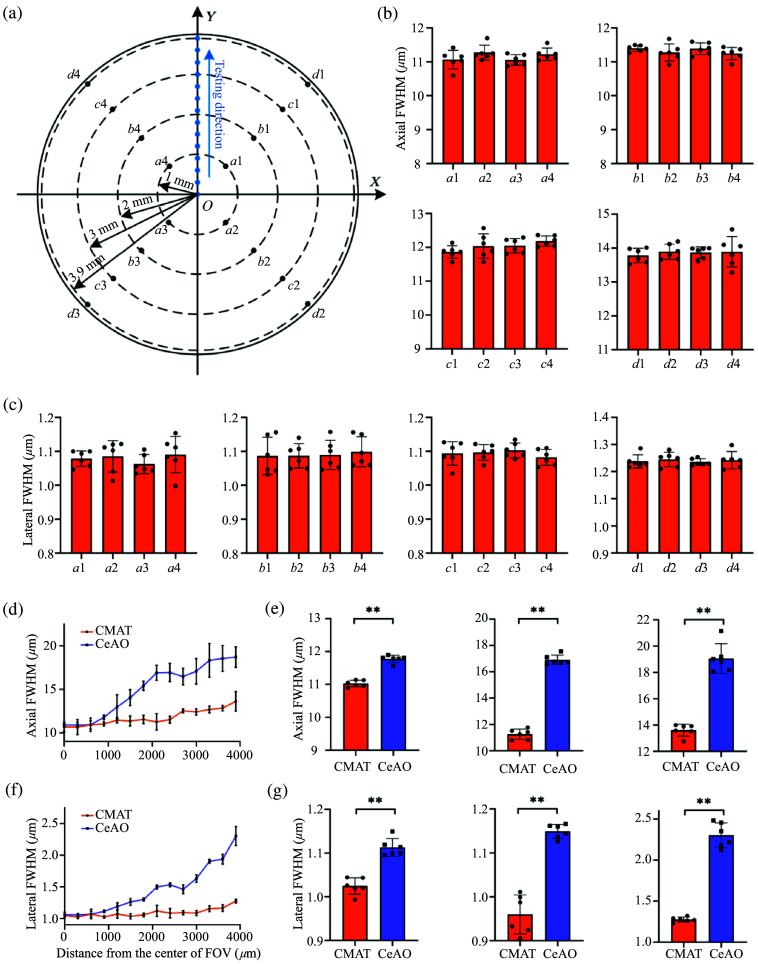
Evaluation of optical resolution utilizing CMAT and CeAO. (a) The uniformity of optical resolution under the CMAT condition, and optical resolution measurements were taken at 14 test sites spaced 300  μm apart across the 8 mm FOV. (b) Axial resolution at 1, 2, 3, and 3.9 mm from the FOV center, n=6. (c) Lateral resolution at 1, 2, 3, and 3.9 mm from the FOV center, n=6. (d) Axial resolution as a function of distance from the central point under CeAO and CMAT conditions. (e) At 900  μm from the center of the FOV, a comparison of axial resolutions between CeAO and CMAT (P<0.01, n=6); at 2100  μm from the center of the FOV, a comparison of axial resolutions between CeAO and CMAT (P<0.01, n=6); at 3900  μm from the center of the FOV, a comparison of axial resolutions between CeAO and CMAT (P<0.01, n=6). (f) Lateral resolution as a function of distance from the central point under CeAO and CMAT conditions. (g) At 900  μm from the center of the FOV, a comparison of lateral resolutions between CeAO and CMAT (P<0.01, n=6); at 2100  μm from the center of the FOV, a comparison of lateral resolutions between CeAO and CMAT (P<0.01, n=6); at 3900  μm from the center of the FOV, a comparison of lateral resolutions between CeAO and CMAT (P<0.01, n=6).

We used 0.5  μm fluorescent beads to validate our method. First, the uniformity of optical resolution across the entire FOV was evaluated under the CMAT method. Measurements were taken at four positions located on each of four concentric circles with radii of 1, 2, 3, and 3.9 mm from the center of FOV [Fig. 2(a)]. As shown in Fig. 2(a), the center of the FOV is designated as point O, with test points selected at 300  μm intervals along the Y-axis. Consequently, a radius of 4 mm includes 14 test points in total, including the central point. We evaluated the performance of the CMAT and Central-AO (CeAO) methods at each test point, with results shown in Figs. 2(d) and 2(e). CeAO is a conventional AO method that performs wavefront phase correction at the central point O. Furthermore, we conducted a comparative analysis of the fluorescence intensity of 10-μm beads under both CMAT and CeAO conditions at various positions located at different distances from the center of the FOV (see Fig. S3 in the Supplementary Material). At each test site, three-dimensional imaging of the fluorescent beads was conducted with an axial step size of 0.27  μm. For each axial step, 10 images were recorded and averaged to reduce measurement errors. Based on the system hardware, the pixel size is ∼0.0542  μm. Accordingly, the fluorescence images, which are 1200×1200  pixels, correspond to an FOV of $65\times 65\;\mu m^{2}$. For each test site, six fluorescent beads were randomly selected. Using a custom MATLAB algorithm, Gaussian fitting was applied to determine the FWHM of each bead. The mean ± *SD* of the lateral and axial FWHM values for the six beads was then calculated for that site.

### 2.3 Animal Setup and Surgery

All experimental procedures involving mice were conducted in the animal facility of the Suzhou Institute of Biomedical Engineering and Technology (SIBET) under protocols reviewed and approved by the Institutional Animal Care and Use Committee (IACUC), following international standards for animal welfare. The animals were group-housed under a 12-h light/dark cycle (lights on at 7:00 AM). All experimental procedures were conducted in compliance with animal ethics regulations and approved by the SIBET Animal Protection and Utilization Committee.

The study used transgenic mouse lines Glutamic acid decarboxylase (GAD); GAD67-GFP knock-in mice),^35^ Thy1-EGFP mice (Jackson Labs stock 007788),^36^ and Thy1-GCaMP6s (C57BL/6J-Tg(Thy1-GCaMP6s)GP4.3Dkim/J, Jackson Labs stock #025393 and #024275).^37^ For *in vivo* optical imaging, a cranial window was surgically prepared with careful procedures: (1) a custom metal headplate was first attached to the skull using Superbond C&B dental cement (Sun Medical, Japan); (2) after cement hardening, a 5 to 8 mm diameter craniotomy was made, and the edges were carefully polished before placing the coverslip; (3) the window was sealed with Tetric EvoFlow T1 dental cement. Postoperative care included twice-daily intraperitoneal injections of cefazolin (North China Pharmaceutical, 500  mg/kg) for three days to prevent infection. All surgical procedures were conducted under the supervision of SIBET’s Animal Ethics Committee. The protocol strictly adheres to international standards for animal research ethics. This surgical method provides a stable optical window for subsequent *in vivo* imaging while ensuring animal welfare through detailed postoperative care. The cranial window permits long-term imaging of neural activity while maintaining physiological stability.

### 2.4 Imaging Cell Bodies in GAD Mice

We conducted validation experiments on GAD mice with 6 mm cranial windows to compare the fluorescence excitation efficiency of CMAT versus CeAO. The mice were anesthetized using an RWD isoflurane system following a specific protocol: initially, 5% isoflurane was administered in an anesthesia chamber for 2 min until full anesthesia was achieved. The anesthetized mice were then positioned under the objective using a custom-made skull fixation frame. During the experiments, anesthesia was maintained with a nose cone delivering 1.5% to 2% isoflurane. Throughout the imaging session, core body temperature was kept constant at 37°C using a heating pad.

### 2.5 Imaging Dendritic Spines in Thy1-EGFP Mice

We utilized a Thy1-EGFP mouse to observe neuronal structures, with a cranial window of 5 mm in diameter. To enhance visualization of sparser samples, the craniotomy was positioned slightly toward the right side of the skull. As delineated in Sec. 2.4, anesthesia was sustained throughout the experiments using the RWD gas anesthesia system under consistent conditions.

### 2.6 Imaging Neuronal ${\mathrm{Ca}}^{2+}$ Activity in Awake Thy1-GaCMP6s Mice

We utilized a Thy1-GCaMP6s mouse with an 8-mm cranial window to verify whether CMAT enhances its ability to capture dynamic biological signals. During the experiment, the mouse remained awake. It was positioned on a carbon fiber annular disk mounted on an air-floating platform, levitated 0.5 to 1 mm above the platform surface. Although the mouse’s head was secured via cranial clamps, limb movements could translate or rotate the disk, creating a perception of relative motion that maintained the animal in a relatively natural activity state within this virtual environment.

### 2.7 Data Analysis

Data analysis was conducted utilizing specialized software, including LabVIEW 2018 (National Instruments), ImageJ (FIJI, NIH), Prism 8.0 (GraphPad), MATLAB 2018b (MathWorks), PyCharm 2024.3 (JetBrains), and Suite2p.

All data are reported in the form of mean ± *SD*. Data analysis methods included the Wilcoxon test for paired samples and the Mann–Whitney test for all other comparisons, as specified in the figures. A p-value of less than 0.05 was considered statistically significant, with significance levels denoted as follows: * for P<0.05, ** for P<0.01, and *** for P<0.001. Nonsignificant results were labeled as “ns.” All statistical analyses were performed using GraphPad Prism software (version 8.0). No additional tests were employed to verify whether the data met the assumptions of the statistical methods. Detailed statistical parameters for each experiment are provided in the figure notes.

The Gaussian fitting of 0.5-μm fluorescent beads in both XY and XZ planes was performed using MATLAB to determine the resolution. Morphological data obtained from GAD and Thy1-GCaMP6s mice were processed in FIJI for averaging, projection, and threshold adjustment. The ROI Manager function in ImageJ was employed to obtain mean pixel values within circular ROIs and pixel values along lines, with the data saved in Excel format. To correct for brain motion artifacts (x−y movement) caused by animal respiration and heartbeat, we utilized the TurboReg plugin in ImageJ to align images in the XY plane. Neuronal activity in Thy1-GCaMP6s mice was identified and segmented using Suite2p after motion correction.^38^ ${\mathrm{Ca}}^{2+}$ responses from identified neurons were expressed as relative fluorescence changes (ΔF/F), calculated as $\Delta F/F=(F-F_{0})/F_{0}$, where $F_{0}$ represents the baseline fluorescence, taken as the 25th percentile of the entire fluorescence recording. According to the settings of Suite2p, the true signal F was computed as the neuronal signal minus 0.7 times the neuropil signal. The signal-to-noise ratio (SNR) of the cells was calculated using the formula SNR = mean $(\Delta F/F)/F_{\mathrm{std}}$, where the mean of ΔF/F was derived from the interval containing the top 10% of signal values, and $F_{\mathrm{std}}$ denotes the standard deviation of noise in the relative fluorescence changes, which was calculated using data below the 90th percentile of the relative fluorescence signal.

## 3 Results

### 3.1 CMAT versus CeAO: Optical Resolution Benchmarking

Figure 2(a) illustrates the uniformity of optical resolution under the CMAT condition; it also shows how optical resolution varies with the position relative to the FOV center under both CMAT and CeAO conditions. The results indicated a largely homogeneous optical resolution distribution across the entire FOV [Figs. 2(b) and 2(c)]. As the distance increases, CeAO fails to adequately compensate for aberrations at the FOV edges, leading to rapid degradation in resolution. Conversely, CMAT effectively overcomes this limitation, maintaining high lateral and axial resolution even at the FOV edges [Figs. 2(d) and 2(e)]. Using GraphPad 8, fluorescent beads were assessed at three positions: 900  μm (significant resolution difference), 2100  μm (middle of FOV), and 3900  μm (field edge) from the center of FOV. Statistical analysis revealed that at 900  μm, CMAT improved axial resolution by ∼6% (P<0.01, n=6) and lateral resolution by ∼9% (P<0.01, n=6) compared with CeAO [Fig. 2(f)]. At 2100  μm, CMAT improved axial resolution by ∼33% (P<0.01, n=6) and lateral resolution by ∼25% (P<0.01, n=6) compared with CeAO. At 3900  μm, where system aberrations cause severe resolution loss, CMAT still provided notable improvements: axial resolution by around 37% (P<0.001, n=6) and lateral resolution by ∼80% (P<0.001, n=6) [Fig. 2(g)]. Despite this, a degradation compared to the system’s optimal resolution at the center persisted. Under CMAT, the resolution at the FOV edge was measured as 13.62±0.458  μm (axial) and 1.276±0.03  μm (lateral), whereas at the FOV center, it was 10.681±0.227  μm (axial) and 1.045±0.012  μm (lateral), based on n=6 measurements. These results indicate that CMAT substantially enhances resolution at the FOV edges.

### 3.2 Morphology of Neurons in GAD Mice

**Fig. 3:**
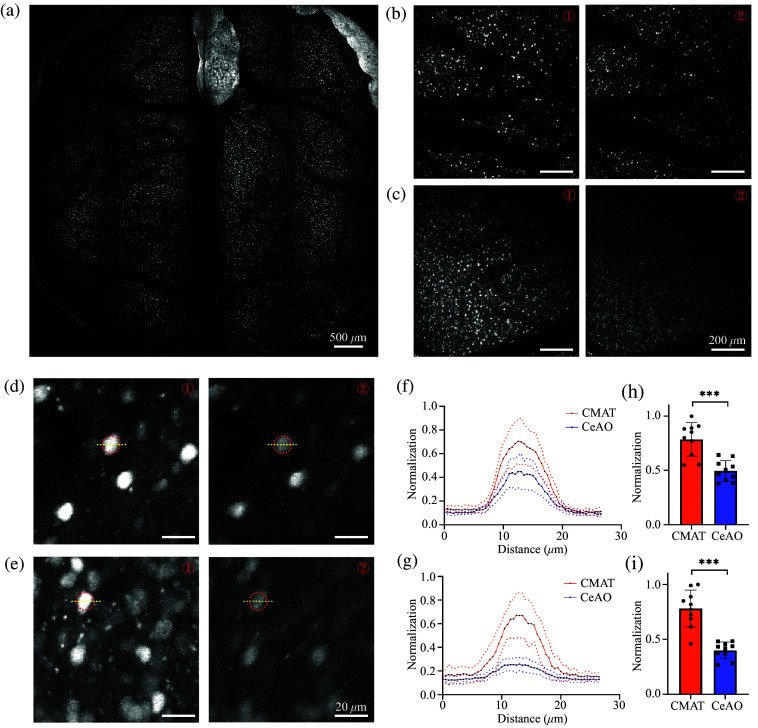
Comparison of fluorescence intensity under CeAO and CMAT conditions in GAD mice. (a) A comprehensive scan of a 6 mm diameter cranial window utilizing CMAT. (b) At 2000  μm from the center of the FOV, a area was scanned: ①shows the GAD image obtained with CMAT, ② with CeAO. (c) The same method was applied at 3900  μm from the center of the FOV. (d) Randomly selected identical neuronal somata in (b)-① and (b)-② were circled in red for mean intensity calculation, with yellow lines indicating cross-sectional profiles. (e) Similarly circled neuronal somata from (c). (f) Normalized intensity profiles from the yellow lines in (d) (n=10). (g) Normalized intensity profiles from yellow lines in (e) (n=10). (h) Normalized mean intensity from the red circles in (d) (P<0.001, n=10). (i) Normalized mean intensity from the red circles in (e) (P<0.001, n=10).

Using the ROI function to select the test area, we conducted z-stack imaging at regions 2000 and 3900  μm from the center of the FOV. The ROI object-space field size was $1\times 1\;mm^{2}$, with a pixel size of 0.5  μm, a Z-axis step size of 5  μm, 10-frame averaging per layer, and a recording depth of 320  μm. The mean ± *SD* statistical method was applied to calculate and analyze ROI data from N=10 neuronal somata, using the Wilcoxon method to compare fluorescence intensity differences betweenCMAT and CeAO conditions. Figure 3(a) displays the morphology of GAD mouse neurons from a global scan. Using ImageJ, maximum intensity projections were generated for the ROI data, showing neuronal somata projections at 2000  μm under CMAT [Fig. 3(b)-①] and CeAO [Fig. 3(b)-②] conditions, as well as at 3900  μm under CMAT [Fig. 3(c)-①] and CeAO [Fig. 3(c)-②] conditions, with identical display thresholds. Identical neuronal somata at 2000 and 3900  μm under both CMAT and CeAO conditions were manually segmented for analysis, [Fig. 3(d)] extracted from Fig. 3(b), Fig. 3(e) extracted from Fig. 3(c). Figures 3(f) and 3(g) show the cross-sectional intensity profile statistics (n=10) for neuronal somata within the yellow ROIs in Figs. 3(d) and 3(e), respectively. Under CMAT conditions, image contrast was significantly enhanced, with greater improvement observed at larger field distances. Figures 3(h) and 3(i) display the mean whole-cell brightness values (n=10) for neuronal somata within the red ROIs in Figs. 3(d) and 3(e), respectively. With CMAT, fluorescence intensity increased by ∼37% (P<0.001, n=10) at 2000  μm and by ∼95% (P<0.001, n=10) at 3900  μm from the center of FOV compared with CeAO. These results showed that the improvement in fluorescence intensity provided by CMAT became more pronounced with increasing distance from the center.

### 3.3 Neuronal Morphology Recording of Thy1-EGFP Mice

**Fig. 4:**
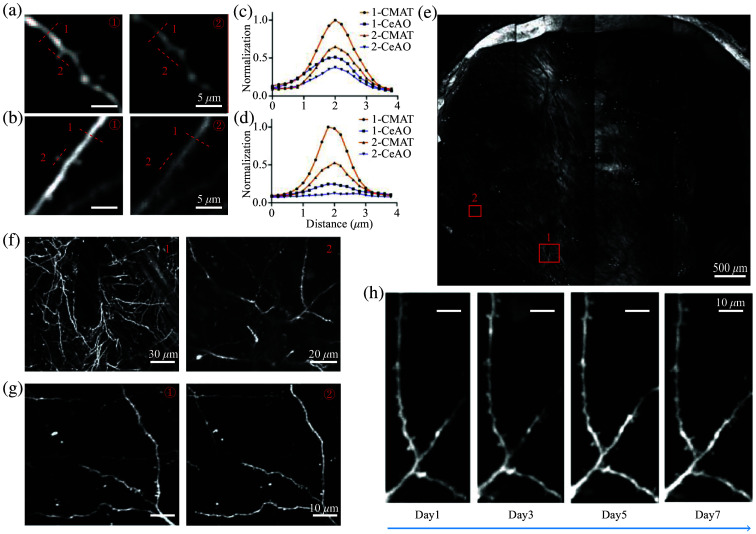
Comparison of image detail contrast in Thy1-EGFP mice under CeAO and CMAT conditions. (a) At 2000  μm from the center of FOV: ① shows spine morphology under CMAT, and ② shows the same location under CeAO. (b) Same comparison at 3900  μm from the center of FOV. (c) Normalized intensity profiles along red lines in (a). (d) Normalized intensity profiles along red lines in (b). (e) Single layer from whole-brain z-stack scan (5 mm cranial window) using CMAT. (f) Magnified views of red boxes in (e) (Image 1 and 2). (g) ①: a region from whole-brain z-stack scan; ②: CMAT scan of corresponding ROI in **I**. (h) Long-term tracking of spine structures using CMAT’s ROI scanning function.

To assess whether CMAT could improve the contrast of fine structural details compared with CeAO, we selected positions at 2000 and 3900  μm from the center of the FOV. Using the ROI function, we acquired a $190\times 190\;\mu m^{2}$ area with a pixel size of 0.2  μm for Z-stack imaging (1  μm step size, 10-frame averaging per layer, 55  μm depth). Figure-① shows the CMAT condition, whereas Fig.② shows the CeAO condition. At 2000  μm from the FOV center, measurements of normalized cross-sectional intensity profiles of neuronal synapses indicated that CMAT provided higher contrast of fine structural details [Fig. 4(c)] and better visualization of neuronal morphology [Fig. 4(a)]. At 3900  μm from the FOV center, the spines were barely visible under CeAO, whereas CMAT enabled clear observation [Fig. 4(b)], demonstrating significantly enhanced contrast of fine structural details [Fig. 4(d)].

Furthermore, to evaluate CMAT performance in large-scale scanning, we imaged the entire $5\times 5\;mm^{2}$ craniotomy area. To ensure clear spine visualization, we set the scan to 16,000×18,000  pixels (0.226  μm/pixel) at 4  s/frame. Z-stack imaging covered 120  μm depth with 0.3  μm steps and 2-frame averaging. Maximum intensity projection of 4.5  μm axial range is displayed in Fig. 4(e), with two enlarged regions clearly showing spine structures [Fig. 4(f)]. Another spine-containing region from the full-FOV scan was enlarged [Fig. 4(g)-①], then recentered for high-resolution 800×800  pixels rescan [0.163  μm/pixel, Z-stack reconstructed in Fig. 4(g)-②]. Comparison confirmed that CMAT enables clear visualization of fine neuronal structures throughout the full field.

To verify the stability of CMAT, we conducted an extended monitoring period of a small ROI over seven days, during which dendritic spines remained distinctly observable [Fig. 4(h)].

These results confirmed that CMAT significantly improves the contrast of fine structural details compared with CeAO and operates stably.

### 3.4 Assessment of ${\mathrm{Ca}}^{2+}$ Signals

**Fig. 5:**
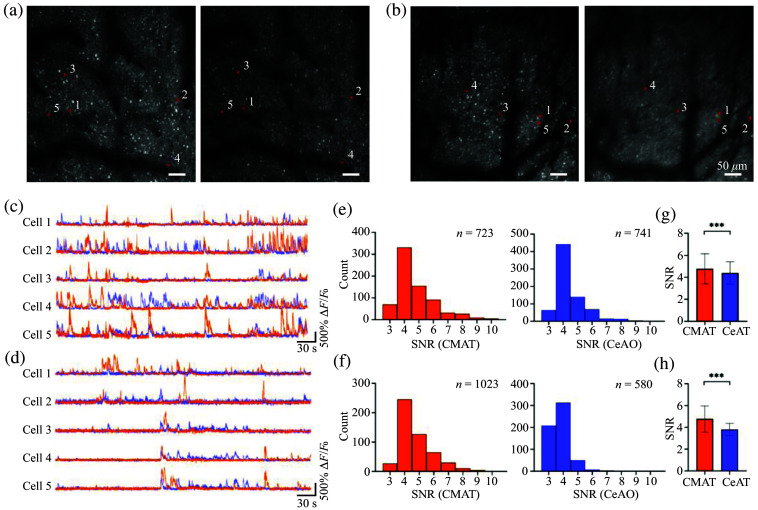
Comparison of ${\mathrm{Ca}}^{2+}$ signals in Thy1-GCaMP6s mice under CeAO and CMAT conditions. (a) At 2000  μm from the center of FOV: ① shows the ${\mathrm{Ca}}^{2+}$ signal morphology under CMAT, ② shows the same location under CeAO. (b) Same comparison at 3900  μm from the center of FOV. (c) Fluorescence fluctuations of five overlapping cells from (a)-① and (a)-②. (d) Fluorescence fluctuations of five overlapping cells from (b)-① and (b)-②. (e) SNR distribution of cells in (a). (f) SNR distribution of cells in (b). (g) Statistical comparison of SNR in (e), P<0.001. (h) Statistical comparison of SNR in (f), P<0.001.

We performed recordings of Thy1-GCaMP6s mice at 2000 and 3900  μm from the center of FOV using the ROI function, with condition ① representing CMAT recordings and condition ② representing CeAO recordings [Figs. 5(a) and 5(b)]. The ROI size was set at 1000×1000  pixels (0.5  μm/pixel) with an acquisition rate of 15 Hz, recording each region for 300 s. Following automated cell identification conducted using Suite2p and subsequent manual exclusion of false-positive signals, five overlapping cells were randomly selected from Figs. 5(a) and 5(b) to analyze their fluorescence fluctuations. The analysis revealed generally higher signal peaks under CMAT conditions [Figs. 5(c) and 5(d)]. Statistical analysis of SNR distribution for all cells in Figs. 5(a) and 5(b) is plotted in histograms of ${\mathrm{Ca}}^{2+}$ signal SNR histograms at 2000  μm [Fig. 5(c)] and 3900  μm [Fig. 5(d)] under both CMAT and CeAO conditions. Figures 5(a) and 5(e) demonstrate superior SNR performance under CMAT, while Figs. 5(b) and 5(f) show that CMAT not only enhances SNR but also recovers signals otherwise masked by noise. At 2000  μm from the center of FOV, SNR was 4.78±1.37 (CMAT) versus 4.41±1 (CeAO), representing ∼9% improvement [Fig. 5(g), P<0.001]. At 3900  μm from the center of FOV, SNR was 4.77±1.2 (CMAT) versus 3.8±0.57 (CeAO), representing ∼23% improvement [Fig. 5(h), P<0.001]. These results suggested that CMAT can significantly improve the SNR of neural ${\mathrm{Ca}}^{2+}$ signals compared with CeAO.

## 4 Discussion

In this study, we introduced a multipoint wavefront correction technology capable of extending the effective imaging FOV of the two-photon mesoscope from 6×6 to $8\times 8\;mm^{2}$. This represents nearly a three-fold increase in effective imaging area compared to current state-of-the-art two-photon mesoscopes, usually with a maximum FOV of $∼5\times 5\;mm^{2}$ and at the same level of NA∼0.5.^22^^,^^24^^,^^26^ Although this method is theoretically applicable to even larger FOVs, current physical constraints of objective lenses inhibit further expansion, as increased scanning angles would cause the incident laser spot to surpass the lens diameter, leading to energy cutoff at the sample plane.^39^ Furthermore, aberrations intensify with increasing distance from the center of the FOV. Most adaptive optics systems possess a finite dynamic range for correction, beyond which aberrations cannot be effectively compensated [see Fig. S2(b) in the Supplementary Material].

Furthermore, for full-FOV scanning, we developed a 4×4 matrix scanning protocol with automatic optimal AO mode matching. This approach more effectively sustains high optical resolution throughout the entire FOV compared with using only a single central correction point. Building upon this technology, we established a 16,000×18,000  pixel acquisition mode that preserves image resolution during large-FOV scanning, enabling observation of fine neural structures across entire brain regions (Sec. 3.3). According to Fig. S1 in the Supplementary Material, the effectiveness of aberration correction diminishes with increasing distance from the center of the FOV, leading to pronounced image degradation in peripheral sub-regions. Although dividing the scanning field into a 5×5 matrix or more subregions enhances image quality across the FOV through the use of additional compensation points, it also results in a multiplicative increase in total scanning time. This trade-off between imaging speed and quality must be selected based on the specific application. In this experiment, as in most biological studies, full-field scanning was employed primarily for the macroscopic localization of samples. Therefore, a 4×4 subregion configuration was chosen.

Therefore, the ROI function served as the primary method in this experiment. The ROI function automatically applies the optimal adaptive optics parameters based on the centroid position of the selected area to achieve the best optical performance for that region. By selecting the small scanning regions flexibly, it achieves an optimal balance between imaging quality and speed. Even in the peripheral 3.9 mm region, we successfully attained lateral and axial resolutions of 1.3 and 14  μm, respectively.^24^^,^^26^ As demonstrated in Sec. 3.2, CeAO alone leads to both fluorescence intensity reduction and morphological distortion toward field edges, whereas CMAT substantially improves both brightness and structural integrity in peripheral regions. The long-term stability of CMAT is equally essential for chronic imaging studies. As demonstrated in Sec. 3.3, CMAT’s ability to maintain stable tracking of neuronal structures over extended periods is a critical requirement for cellular morphology studies research.^40^^,^^41^ Comparative studies in Thy1-GCaMP6s mice revealed that under CeAO, both SNR and detectable cell counts decreased dramatically toward field edges, likely due to insufficient SNR for Suite2p identification. This limitation was resolved using CMAT.

The CMAT methodology can be tailored to various systems and FOV dimensions. The detailed procedure is outlined as follows: Initially, a DM is employed to execute wavefront correction along a principal axis, thereby establishing the maximum attainable FOV of the system. Considering the radial distribution characteristics of the FOV, initial correction points are strategically sparsely distributed from the center towards the periphery. The effective working distance of each point is determined by measuring variations in optical resolution at different radial positions, defined explicitly as the critical distance at which a statistically significant difference in resolution occurs. For the peripheral regions of the FOV, a more lenient tolerance criterion is employed, where the effective correction range is demarcated by a resolution degradation not exceeding 10% of the optimal value at the correction point. Subsequently, the effective working distances of all points are subjected to statistical analysis to determine the optimal spacing between correction points, as illustrated in Fig. S1 in the Supplementary Material. The maximum operational FOV of the system is then determined based on the position of the outermost correction point and its associated effective correction range. Typically, this maximum operational FOV closely aligns with the initially determined maximum achievable FOV. Finally, correction points are uniformly distributed across the entire FOV according to the calculated optimal spacing, ensuring seamless and complete coverage by the effective correction areas.

To ensure complete coverage of the entire FOV by the effective correction areas, the spatial utilization of these areas must be maximized. As illustrated in Fig. S4(a) in the Supplementary Material, the CMAT scheme exhibits minimal overlap between adjacent correction points. The overlapping area with one adjacent point constitutes only 5.75% of a single point’s effective area, and the total overlap with all surrounding points is 34.5%. In contrast, the N×N layout exhibits significantly larger overlaps of 18.12% with one adjacent point and 72.48% in total [see Fig. S4(b) in the Supplementary Material]. Consequently, regardless of how the absolute size of a single effective area is defined, the CMAT scheme achieves full FOV coverage with fewer correction points per unit area. This advantage becomes particularly significant on the mesoscopic scale.

## 5 Conclusion

The CMAT developed in this study efficiently improves the FOV limitations of the two-photon mesoscopes, extending the effective imaging diameter from the originally designed 6 mm to a practically usable 8 mm (∼50% area increase) while significantly improving image quality. Comprehensive validation demonstrates the following advantages of this technology: (1) improving resolution with 37% and 80% in axial and lateral resolution, respectively; (2) dramatically increasing imaging sensitivity, achieving up to 95% fluorescence signal boost in GAD mouse models; (3) markedly improving image quality, with significantly enhanced fine structural contrast in Thy1-EGFP mice; and (4) optimizing functional imaging performance, showing a 23% SNR increase in Thy1-GCaMP6s mice and detection of more active neurons in peripheral regions. These results indicate that CMAT effectively addresses the critical challenges of resolution degradation and signal loss in large-FOV imaging through innovative adaptive correction methods, providing a reliable platform for fine-scale observation of pan-cortical neural circuits. With broad application potential, this versatile technology can be adapted to various mesoscopic imaging needs, establishing a new paradigm for large-scale, high-resolution imaging in neuroscience research.

## Supplementary Material

nph-25121GR-s01.pdf

10.1117/1.NPh.13.1.015004.s01

## Data Availability

Codes and Data used in this study are available at: https://github.com/cssyyyjl/CMAT-PAPER_DATA.
