# Supplementary material for: Cellular multipoint adaptive technology for two-photon mesoscope
Source: Neurophotonics. 2026 Jan 20;13(1):015004. doi: 10.1117/1.NPh.13.1.015004 (PMC12818465; doi:10.1117/1.NPh.13.1.015004)
Supplement: Supplementary file 1 [file NPh_013_015004_SD001.pdf]

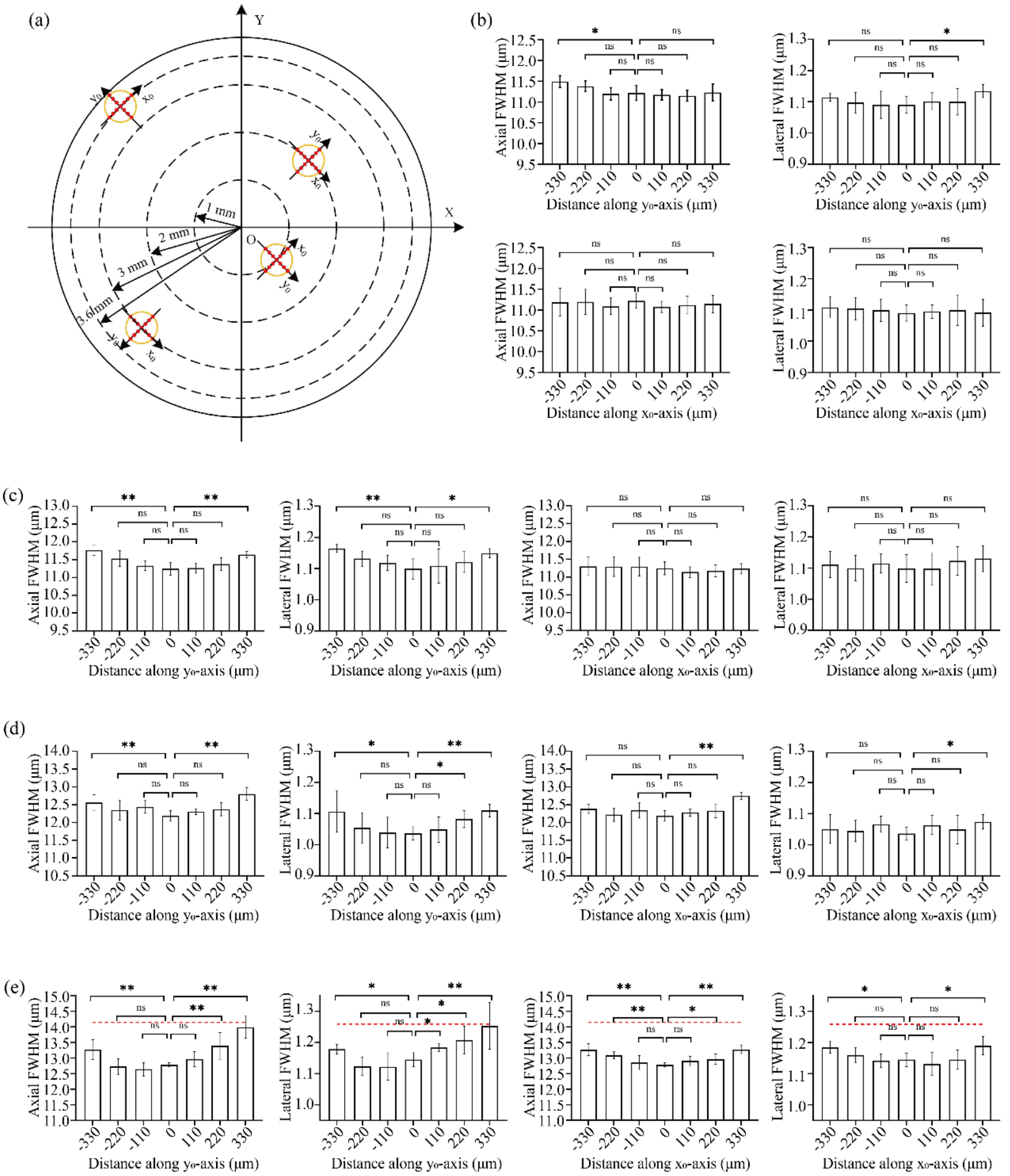

Supplementary Fig. S1 Characterization of correction point spacing. (a) Schematic of the measurement locations. Four positions were evaluated at radial distances of 1, 2, 3, and 3.6 mm from the center of the FOV. At each location, the optical resolution was measured at 13 points arrayed linearly along the x_0- and y_0-axes, covering a range from –330 μm to +330 μm with a uniform spacing of 110 μm. (b-e) Spatial distribution of axial and lateral resolution measured at 1 mm, 2 mm, 3 mm, and 3.6 mm from the FOV center, respectively (n = 6). At 3.6 mm from the center of the FOV, the red line indicates the threshold corresponding to a value 10% above that at the center of this region.

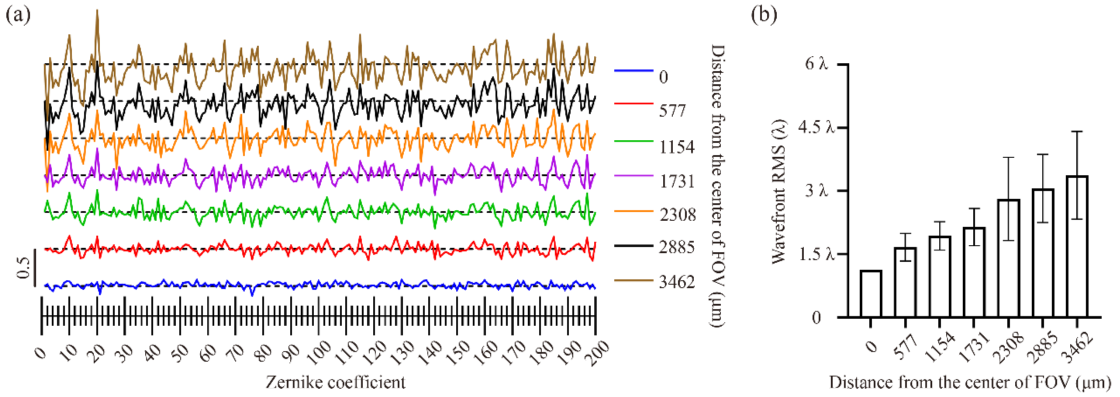

Supplementary Fig. S2 Analysis of aberration correlations. (a) The 200 Zernike coefficients for aberration correction at points located 0, 577, 1154, 1731, 2308, 2885, and 3462 μm from the center of the FOV. (b) Wavefront Root Mean Square (RMS) at points located 0, 577, 1154, 1731, 2308, 2885, and 3462 μm from the center of the FOV, λ=920 nm.

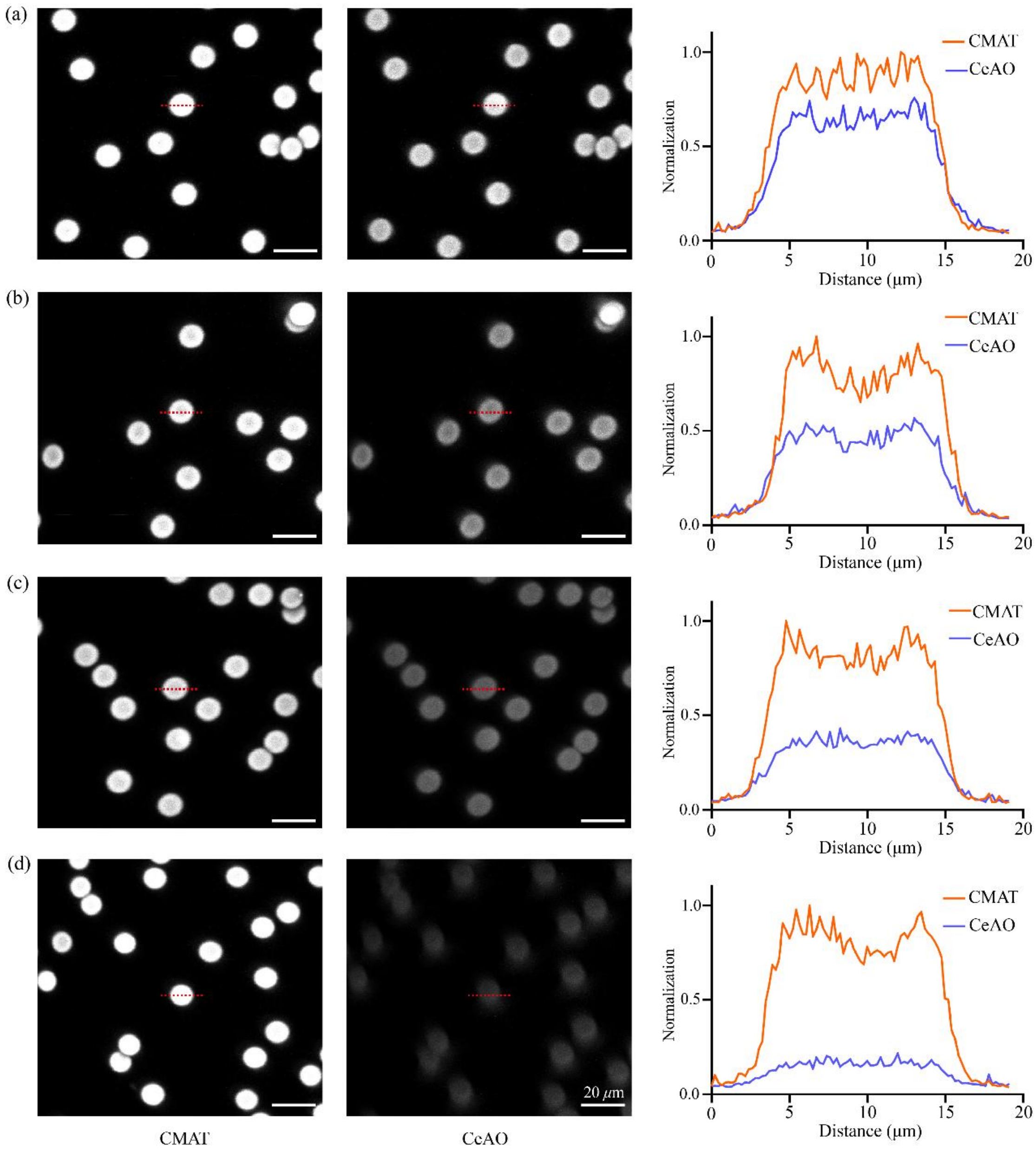

Supplementary Fig. S3 Fluorescence intensity of 10 μm beads: CMAT vs. CeAO across the FOV. (a-d) Left panels: representative images of 10 μm fluorescent beads acquired at 1, 2, 3, and 3.9 mm from the FOV center, respectively. Right panels: corresponding normalized fluorescence intensity profiles along the red lines marked on the beads.

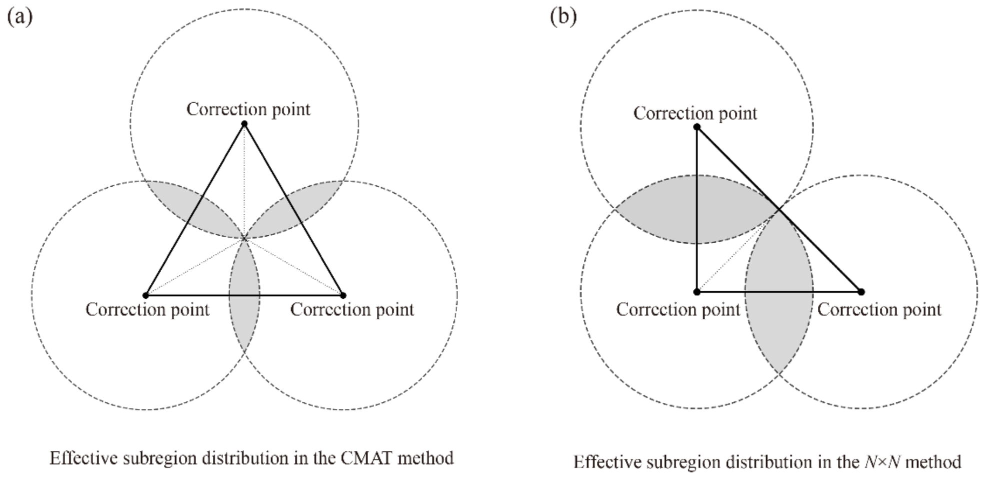

Supplementary Fig. S4 Comparison of different layout strategies for correction points. (a) Distribution of three adjacent correction points and their effective correction areas in the CMAT method. (b) Distribution of three adjacent correction points and their effective correction areas in the $N \times N$ method. The intersection point of the three (effective area) circles is the circumcenter of the triangle formed by the three correction points.
